# Bio-Based Polyethylene Composites with Natural Fiber: Mechanical, Thermal, and Ageing Properties

**DOI:** 10.3390/ma13112595

**Published:** 2020-06-06

**Authors:** Patrycja Bazan, Dariusz Mierzwiński, Rafał Bogucki, Stanisław Kuciel

**Affiliations:** Faculty of Materials Engineering and Physics, Institute of Materials Engineering, Tadeusz Kosciuszko Cracow University of Technology, Al. Jana Pawła II 37, 31-864 Cracow, Poland; dariusz.mierzwinski@pk.edu.pl (D.M.); rbogucki@mech.pk.edu.pl (R.B.); stask@mech.pk.edu.pl (S.K.)

**Keywords:** bio-polyethylene, composites, basalt fiber, flax fiber, wood flour, coconut shell fibers

## Abstract

The study evaluated the possibility of using natural fibers as a reinforcement of bio-polyethylene. Flax, coconut, basalt fiber, and wood flour were used in the work. Strength tests like static tensile test, three-point flexural test, or impact strength showed a positive effect of reinforcing bio-polyethylene-based composites. The effect of water and thermal ageing on the mechanical behavior of composites was assessed. In order to analyze the structure, SEM microscope images were taken and the effect of natural fibers on the change in the nature of cracking of composites was presented. Composites with natural fibers at a content of 12% by weight, resulting in increase of strength and rigidity of materials. The greatest strengthening effect for natural fibers was obtained for the composite with basalt fibers.

## 1. Introduction

The problems of environmental protection, depletion of fossil raw materials (mainly petroleum), and environmental sustainability are important reasons why many producers and scientists undertook intensive research in the field of material engineering, recycling, and technology for the production of new, polymeric, user-friendly materials for the natural environment [1].

Examples of such materials may be bio-composites composed of natural fibers and matrices of synthetic non-biodegradable polymers that only partially biodegrade over time, or composites containing both biodegradable components called green composites [2]. The most popular biodegradable materials include polylactide (PLA), poly (3-hydroxybutyrate-co-3-hydroxyvalerate) (PHBV), and thermoplastic starch. The advantages of biodegradable plastics are their high mechanical properties, but most importantly they are environmentally friendly materials, decomposing into products that occur naturally in nature and they are used in the automotive, construction, and fiber optics industries [3,4,5,6,7].

Polymers as carbon compounds are most often produced from petrochemical sources, but increasingly from renewable sources. Non-biodegradable plastics from renewable resources include the plastics where biomass is used for production of plastics without the biodegradation property. They are mostly made from bioethanol produced from sugar fermentation, biofuel such as polyethylene (bio-PE). Some other examples are polyvinyl chloride(bio-PVC), polyethylene terephthalate (bio-PET), or polypropylene (bio-PP) [8].

The basic monomer of polymer polyethylene is ethylene. Ethanol, which is similar to ethylene, can be produced by fermenting corn or sugar cane, which is why the expression “bio-polyethylene (bio-PE).” It is chemically and physically similar to traditional polyethylene. It is not biodegradable, but can be recycled. Bio-polyethylene is currently produced on an industrial scale from bioethanol from sugar cane. In addition, it is an environmentally friendly material and has similar technical properties and processability of the resin from fossil sources. Therefore, processing green plastic does not require any technical adjustments or new investment in equipment [9].

The continuous increase of requirements for materials, both for general use and construction materials causes the increasingly widespread introduction to the market of petrochemical composite materials with artificial fibers, bio-composites, and green composites. To meet the requirements, especially those regarding environmental changes, natural fibers and particles that do not have a negative impact on the environment are more often used. Natural additives are obtained from renewable sources and also provide fairly good mechanical properties. The most well-known plant fibers are: oil palm, wood, rice straw, sisal, ramie, hemp, doum fruit, bagasse, pineapple leaf, cotton, flax, date palm, rice husk, wheat straw, curaua, coir, jowar, kenaf, bamboo, rapeseed waste, roselle, mesta, banana, abaca, agave, maize, and jute [10,11].

Natural fibers (NF) can be divided into wood fiber and non-wood fiber. Wood is a natural and complex polymer composite that essentially contains cellulose, hemicellulose, lignin, and extracts [12].

Most non-wooded plants are annual plants that develop the full potential of fibers in one growing season. Plant fibers can be in the form of hair (cotton, kapok), hard fibers (pen, sisal), and fiber discs (flax, hemp, jute). There are six types of plant fibers, namely bast fibers (flax, hemp, jute, kenaf, and ramie), leaf fibers (abaca, pineapple, and sisal), seed fibers (coir, cotton, and kapok), straw fibers (corn, rice, and wheat), grass fibers (bagasse and bamboo), and wood fibers (softwood and hardwood) [13].

Flax fibers are considered to be the most important member of the bast family used as a reinforcement in composites because of their unique properties. The natural high strength and stiffness of flax, low elongation to break, make them particularly interesting in composite research. Flax fibers are not continuous compared to synthetic ones, but have a similar structure as composites and they are hierarchically organized. Their macroscopic properties result from their micro and nano-structural level [14,15]. Flax composites were extensively studied. They were introduced to composite materials based on thermoplastics, thermosets, and bio-materials. Yan et al. in his review of composites with flax fiber, presented in detail the advantages and disadvantages of flax fibers, both in the form of cut fibers and fabrics [14]. The results showed that flax as a reinforcement causes an increase in strength properties, however the results were strongly dependent on the fiber geometry, surface treatment, and humidity. Assarar et al. studied the effect of water ageing on composites based on epoxy resin with glass fiber and flax fiber. Studies proved that water ageing has a strong effect on flax fiber composites, and greatly reduces the Young’s modulus and strain at break compared to glass fiber materials [16].

Cheour et al. investigated the effect of water ageing on flax fiber-reinforced epoxy composites and their mechanical and damping properties. The results showed a decrease in flexural modulus and an increase in loss coefficients. It was also confirmed that these changes are virtually reversible for loss factors, but irreversible for flexural modules [17]. Chilali et al. also studied the effect of water on flax fiber composites. Tensile strength tests showed a reduction of approximately 10% in the rigidity of aged composites compared to non-aged materials and indicated a strong effect of water damage behavior [18]. Moudood et al. examined effect of humidity on flax epoxy composites. The results showed that the production of composites with highly wet fabrics causes deformation of finished elements after treatment, and also leads to poor microstructural quality. The moisture content of the fibers reduces the tensile and flexural stiffness but also increases fracture toughness [19]. Dhakal et al. also presented the susceptibility of flax fibers to water. Bending properties tend to decrease as the percentage of moisture absorption increases. Comparison of flexural strength and flexural modulus between dry flax bio-composites and wet flax showed that wet samples lost about 50% of strength and stiffness compared to dry flax samples, hence researches were carried out to improve flax fibers behavior [20]. Stambolis et al. presented research on improved flax fiber, which was 30% less water-sensitive, and composites with those fiber were characterized by maintaining strength and stiffness despite adverse environmental effects [21].

Coconut fibers are obtained from the fibrous husk of the coconut from the coconut palm. Coconut fibers have a high lignin content and therefore a high cellulose content, making them elastic, strong, and very durable. The properties of composites with coconut fibers as well as with flax fibers and other natural fibers strongly depend on the volume, diameter, and length of the fibers, as well as moisture content [22]. Singh et al. evaluated the effect of coconut shell powder with different particle sizes and volumes on the tensile and bending properties of composites based on the epoxy resin matrix. Coconut improves the mechanical properties of epoxy resins but changes of properties are influenced by the volume of fillers [23]. However, in studies provided by Reddy T. on thermoplastic coconut fiber composites, where high density polyethylene (HDPE) was used as a matrix, the mechanical properties were more depended on the fiber length [24]. Siva et al. studied the optimal volume content of fibers. The study examined four volumetric parts, the results showed that with increasing fiber content, the strength properties increase and the resistance to dynamic impact decreases. However, because of its higher density, thickness, and more de-laminations, high fiber volume fraction may not always be good for reinforced polyester composites [25]. Onuegbu et al. presented work on various surface treatments of coconut fibers, studies showed that alkali treatment of coconut fibers significantly improves their tensile properties [26].

Wood flour is obtained from wooden raw material and is characterized by a loose, very fine, and even consistency. In the chemical composition of wood flour we find cellulose in about 50%, lignin in about 20–25%, hemicellulose in 20–25%, and also, among others: resins, waxes, rubber, proteins, and mineral salts. The exact concentration of individual substances depends on the species of trees, soil, and weather conditions in the area where the trees grow. The influence of wood flour size on mechanical properties and density of HDPE composites was investigated. The results showed that the wood particle size had a significant effect on wood plastic composite (WPC) properties. The change in the number of meshes has a great effect on the flexural modulus, tensile modulus, and impact strength, however, it has little effect on flexural and tensile strength [27].

Thermoplastics commonly modified with wood flour include work on polypropylene composites with wood flour provided by Sheshmani et al. and Ichazo et al. [28,29], high density polyethylene, and recycled high density polyethylene presented by Hamzeh et al. and Nourbakhsh et al. [30,31]. Despite the advantages, the use of wood in thermoplastic materials has some problems; limitation of wood thermal stability, difficulties in obtaining good dispersion of filler, and weak interphase adhesion. This is due to the natural incompatibility between hydrophilic polar wood fibers and hydrophobic, non-polar thermoplastics. This phase mismatch causes a poor interface between the wood filler and the polymer matrix. In addition, the strong interaction of wood-wood resulting from hydrogen binding and physical entanglement reduces the dispersion of fillers in the viscous matrix.

Recently, basalt fibers (BF) has appeared as a new type of NF. They are widely used in the area of reinforced polymer materials. BF, which are similar to chemical composition of glass fibers, have interesting properties, including high melting points (in the range from 1350 to 1700 °C), high stiffness, excellent heat resistance, and excellent vibration insulators. In addition to these promising properties, BF are relatively cheaper than carbon reinforcement, non-toxic, and natural, which undoubtedly makes them a good alternative to artificial reinforcement polymer materials [32,33,34].

The growing interest in biobased polymer forces researches and manufacturers to work on new composites containing bio-matrix and natural fibers. Serra-Parareda et al. presented research on polyethylene composites with barley straw as an alternative to using agricultural waste. The barley straw content ranged from 15–40% by weight. Strength results showed the fiber has a high ability to provide strength and rigidity for such composites [35]. Castro et al. used curaua fibers as a reinforcement for high density bio-polyethylene. The presence of curaua fibers improved some properties such as bending strength and storage modulus, but they also pointed to the high importance of manufacturing methods and introduction of other modifiers to obtain high-grade composites [36]. In another work by Castro et al. it was confirmed that the introduction of castor and canola oils as a potential compatibilizer for bio-Pe composites with curaua fiber additionally improves the strengthening effect [37]. Kuciel et al. introduced natural additives into bio-polyethylene (HDPE and LDPE) as an additive, such as wood flour, kenaf fibers, cellulose power, and tuff particles. The introduction of natural particles does not affect the results of tensile strength, however, the use of wood flour and kenaf fibers increases the stiffness several times. The introduction of natural particles causes an increase in water absorption that affects the surface quality of the tested materials [38]. Tarres et al. presented the bio-based polyethylene matrix with thermomechanical pulp (TMP) fibers. In addition, the effect of melamine anhydride on mechanical properties was investigated. Studies pointed an increase in the mechanical properties of the tested composites, especially with the addition of a compatibilizer. Researchers proved that thermomechanical pulp fibers facilitate 3D printing and provide products with high strength properties [39].

This work presents new composites based on bio-polyethylene with natural fibers as the first stage of research on hybrid connections of natural fibers. Knowledge of the mechanisms of strengthening natural fibers such as flax fiber, coconut, wood flour, and basalt fibers posed at a further stage to use the full potential of hybrid reinforcement for the same fibers.

## 2. Materials and Methods

### 2.1. Materials

In this work composites based on a high density bio-polyethylene (Green PE SHC7260 produced from renewable source (sugarcane-based ethanol), Braskem, Brazil) were investigated. The standard dog bones samples and bars were made at Cracow University of Technology using KM 40-125 Winner Krauss Maffei. The temperatures in the subsequent zones ranged from 170 °C to 200 °C, injection pressure 1300 bar and the injection speed was set to 60 mm/s. As a reinforcement basalt fiber (BF), (KV02M chopped strand series with diameter of 13 µm, length about 3.2 mm, Kemenny Vek, Dubna, Russia), coconut shell fibers (Coco MLD 2 mm with diameter of 100 µm and length about of 2 mm, Procotex S.A., Moeskroen, Belgium), flax fibers (Flax MLD 2 mm with diameter of 50–70 µm and length about of 2 mm, Procotex S.A., Moeskroen, Belgium), and wood flour (Lignocel BK 40/90 from soft wood (spruce) with particle size 300–500 µm J. Rettenmaier & Söhne Company, Rosenberg, Germany) were used. As a compatibilizator Scona TPPP 9112 FA was used (BYK, Altana, Germany). Composites with a content of 6 and 12 percent by weight were prepared to analyze the reinforcement with natural fibers as a basis for testing hybrid composite materials containing a combination of natural fibers in the next stage of research. Manufactured materials for the experiment are described below in Table 1.

### 2.2. Characterization of Natural Fibers

As described above natural fibers can be divided into wood fiber and non-wood fiber. Table 2 and Table 3 present chemical composition of the used fibers: flax fibers, coconut shell fibers, wood particles, and basalt fibers and their mechanical properties [11].

**Figure 1:**
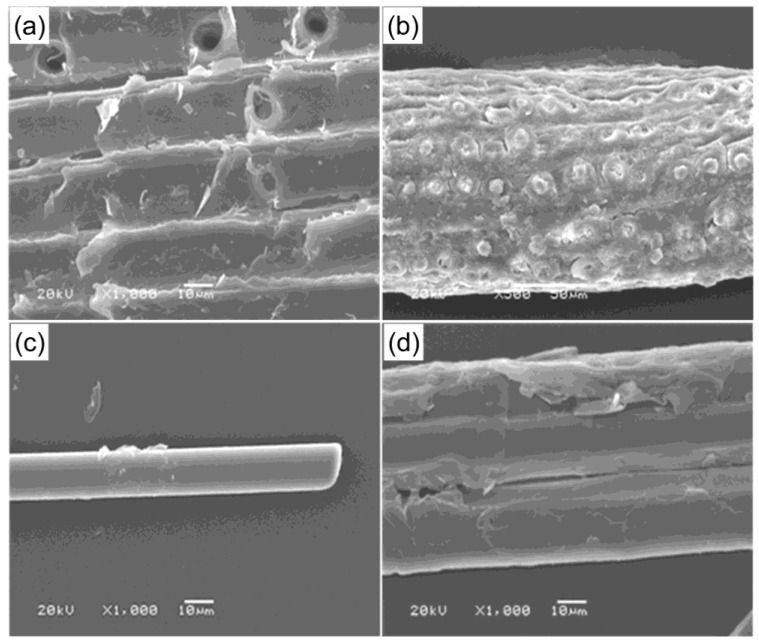
Scanning electron microscopy (SEM) images of surface of natural fibers: (**a**) wood flour, (**b**) coconut shell, (**c**) basalt fiber, (**d**) flax fiber.

Microstructures of fibers used in this work were taken and are presented in Figure 1. Figure 1a presents microscopic picture of wood flour. As with most natural materials, wood anatomy is complex. Wood is porous, fibrous, and anisotropic. Wood consists mainly of hollow, spindle-shaped cells, which are arranged parallel to each other along the trunk of the tree. These fibers are heavily cemented together and form a structural element of wood tissue. The length of wood fibers is variable, but on average about 250–500 µm. The fiber diameters are usually about 15–45 µm. When wood is reduced to wood flour, the resulting particles are actually bundles of wood fibers rather than individual fibers [44]. Figure 1b shows unprocessed coconut fiber. Fiber morphology shows surface roughness. The increased surface roughness of the coconut fiber is the result of higher amount of exposed cellulose. Figure 1c displays basalt fiber. The surface of the fiber is smooth and plane, without visible damage to the fiber. The structure of flax fiber is quite complex. Flax fiber is characterized by multiwalled anatomy which can been seen in the Figure 1d.

### 2.3. Method of Testing

Basic physical and mechanical tests of bio-polyethylene and its composites were carried out.

#### 2.3.1. Physic-Mechanical Characterization

Density was measured by the hydrostatic method by scale RADWAG WAS 22W (Radom, Poland). Static tensile test (PN-EN ISO 527-1:20100) and the three-point flexural test (PN-EN ISO 178:2011) were carried out with a MTS Criterion Model 43 universal testing machine (MTS System Corp., Eden Prairie, MN, USA), using the MTS axial extensometer. The test speed was set to 10 mm/min. Charpy impact test (PN-EN ISO 179-1:2010) was examined on unnotched specimens using a Zwick HIT 5.5P (Zwick Roell Group, Ulm, Germany). In order to determine the initial mechanical hysteresis loops with forced displacement cyclic load and unload tests were investigated using the MTS Criterion 43 universal testing machine with the MTS software TestSuites 1.0 to analyze the dissipation energy. The speed of forced displacement specimens was 100 mm/min, which is translated into a low frequency of cycles and allows to visualize viscosity phenomena. The values were obtained from an average at least of five specimens.

#### 2.3.2. Water Absorption and Water Diffusion Coefficient

Water absorption was carried out according to ASTM D570-98 standard. The specimens were periodically weighed using an electronic weighing balance (RADWAG WAS 22W). Water absorption was calculated using the following equation:

(1) $$W\%=\frac{W_{n}-W_{0}}{W_{0}}\cdot 100$$

where *W*_0_ is the initial weight of the sample, *W*n is the weight of the saturated sample, and *W*% is the percentage increase in weight.

#### 2.3.3. Thermal Aging

Physic-mechanical investigation was repeated after thermal aging. Accelerated ageing tests were conducted in autoclave (Parr Instrument Company, Moline, IL, USA) according to the EN ISO 2440 standard. At first, samples were conditioned at a temperature of 23 ± 2 °C and a humidity equal to 50 ± 5% for 24 h. After the conditioning process, samples were placed in an autoclave where the temperature was set up to 120 °C, the humidity was 100%, and the pressure was 0.3 MPa. The ageing process lasted for 144 h.

#### 2.3.4. Scanning Electron Microscopy (SEM)

The microstructure pictures were made on tensile-test fracture surfaces covered by gold layer to avoid electrostatic charging during SEM analyses. The micrographic images were taken in high vacuum mode with 10 kV accelerating voltage and 13.7 mm working distance using a scanning electron microscope JEOL JSN5510LV (JEOL Ltd., Tokyo, Japan).

#### 2.3.5. Short-Time Relaxation Test

Composites based on bio-polyethylene were subjected to the relaxation process, 2 mm deformation was applied with a forced speed of 1 mm/min, and then the stretching process was stopped and changes in forces needed to maintain the deformation at 2 mm were recorded for 3 h. The tests were carried out on a Shimadzu AGS-X testing machine, with a measuring range up to 1 kN with Trapezium software.

## 3. Results and Discussion

### 3.1. Mechanical Investigation of Composites

Knowledge of basic strength properties is necessary for modeling and designing construction materials. The properties of the composite depend on many factors such as: matrix properties, fiber properties, content of reinforcement, geometry and orientation of the fibers, adhesion between composite components, manufacturing conditions etc. Each of the above-mentioned factors is extremely important and also depends on other factors. In order for the composite material to fulfill its task, there must be a proper connection between components. Adhesion in qualitative terms is the connection of one material to another by means of chemical bonds, intermolecular interactions (Van der Waals forces), hydrogen bonds, ionic bonds, electrostatic interactions, or mechanical bonding of surfaces in contact with each other. Factors affecting the amount of adhesion include: degree of wetting of the fibers; chemical structure of ingredients; the amount of mechanical friction forces at the component boundary; size and direction of shrinkage stress; occurrence and size of physical and chemical forces (adhesion forces), defects in the form of voids, air bubbles, etc., during manufacturing [2]. Table 4 presents the results obtained through basic strength tests.

The results of basic strength tests showed the largest impact of basalt fiber on strength properties, which of course is associated with the high mechanical properties of the fiber. From natural fibers, the increase in tensile strength was provided by flax fiber, with an amount of fibers of only 6% by weight, the gain was small, by about 5%, with a fairly small rise in stiffness about 40%, which is a poor result in relation to wood flour modification (increase in the module by almost 90%); however, the results of materials with a content of 12% by weight of flax fiber caused an increase in strength by 20% and the module more than twice compared to neat polyethylene which is most likely related to the structure of flax fiber. Flax is cellulose fiber, but its structure is more crystalline, owing to which, although it is more fragile, it is much stronger, and its cross-section contains about 40 fibers [45]. In the case of other fibers, doubled content of fillers did not bring about large changes in mechanical properties, which is associated with very poor adhesion of natural fibers and particles to polymer matrices because of the hydrophilicity of fibers and the hydrophobicity of polymers. This is due to the basic fiber components such as cellulose, hemicellulose, and lignin. All these materials contain hydroxyl groups and other oxygen-containing groups that facilitate moisture absorption through hydrogen bonding and this is responsible for dimensional changes because of swelling and shrinkage, affecting mechanical properties [46]. The possible solution to this problem may be surface treatment of fibers with adhesion increasing agents (containing functional groups capable of chemical reactions with functional groups contained in the polymer) or increasing wettability and reducing surface tension.

**Figure 2:**
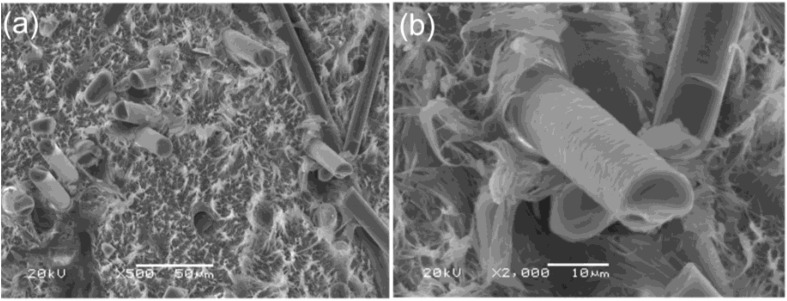
SEM images of bio-polyethylene with basalt fibers (**a**) 500× and (**b**) 2000× magnification.

Microscopic images show the microstructures of composites. Figure 2a,b present composites with basalt fiber. The polyethylene matrix had a fibrous, ductile character, and basalt fibers had a relatively smooth surface. The connection between composite components was purely mechanical without major chemical changes in interphase. The picture also shows holes as a result of the phenomenon of pulling fibers out of the matrix as well as the surfaces of fiber cracks, which indicates the mixed nature of composite damage. Random fiber orientation can also be observed.

**Figure 3:**
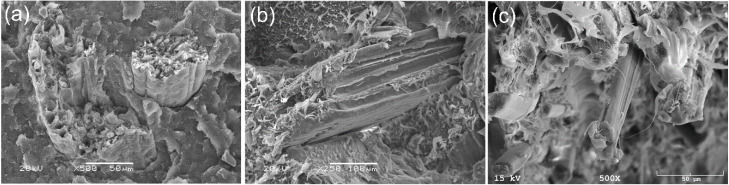
SEM images of bio-polyethylene composites with (**a**) coconut shell fiber, (**b**) wood flour, and (**c**) flax fiber.

Figure 3 shows composites with coconut fiber, wood flour, and flax fiber. The structure of the coconut fiber composite (Figure 3a) was more brittle without plastic extensions. In the case of wood flour composite (Figure 3b), the nature of the cracking was plastic and the developed surface around the wood particles could be observed. Nonetheless, free spaces can be observed between the fiber and the matrix, indicating a lack of chemical connection between the components. The appearance of the microstructure only confirms the results obtained in mechanical tests and indicates the necessity of using coupling agents. Figure 3c presents flax fiber composites. The polygonal shape of flax fibers with sides 5–7 can be seen. The microstructure of the fiber is extremely complex because of its heterogeneous nature along the entire length. The fiber orientation in the compositions was random, and the matrix exhibited a fairly plastic character. A better connection between the ingredients compared to the other compositions is visible.

**Figure 4:**
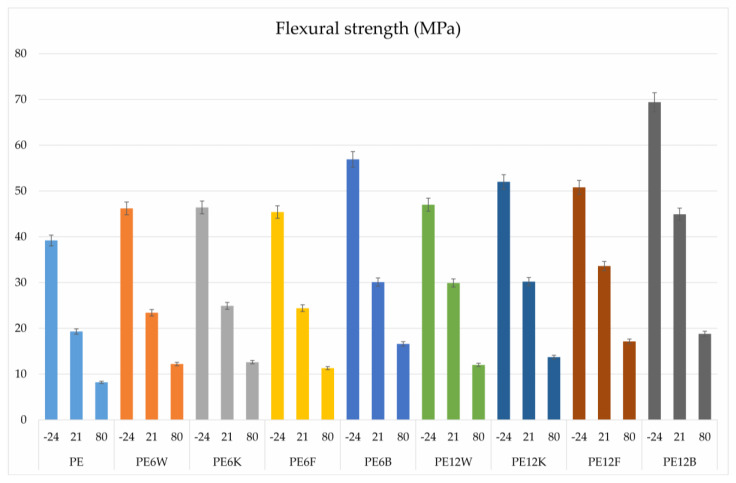
Comparison of flexural strength of tested material.

**Figure 5:**
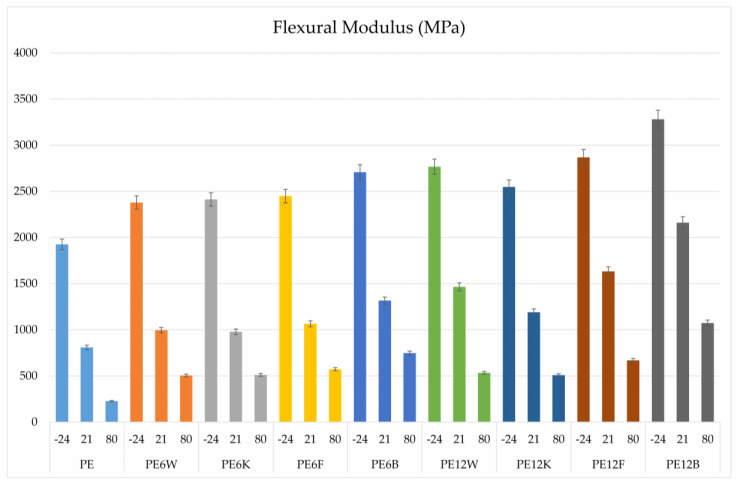
Comparison of flexural modulus of tested material.

Plastics and composites as construction materials are subject to various external factors. An important physical factor for plastics is the exploitation temperature. The behavior of composites at temperatures is significantly influenced by the geometry and volume of fibers. Figure 4 and Figure 5 show the results of a three-point flexural test over a wide temperature range (−24, 21, 80 °C). The results showed a proportional decrease in mechanical properties with increase in temperature, which is associated with a higher mobility of polymer segments at elevated temperatures. The highest results were obtained for the material with basalt fiber. Noteworthy is the fact that in the case of materials working on bending conditions, not much higher content of flour or coconut fiber (12% by weight) can provide similar strength parameters as the material with basalt fiber in an amount of 6% by weight. Analyzing the effect of temperature on bending properties, it was flax fiber that provided the greatest stability of measured parameters, since the decrease with increase in temperature conditions was the smallest for this material.

There are various sources of dissipation energy in fiber-reinforced composites: the viscoelastic nature of the matrix, the phase between the fiber and the matrix, damages, viscoelastic suppression, and thermoplastic suppression. The low dissipation energy value corresponds to the large volumetric strain, which in turn means that the cavitation process is not the main source of dissipation energy. The general point of view for polymers and polymers filled with particles is assumed that dissipation energy mainly comes from matrix deformation [47].

**Figure 6:**
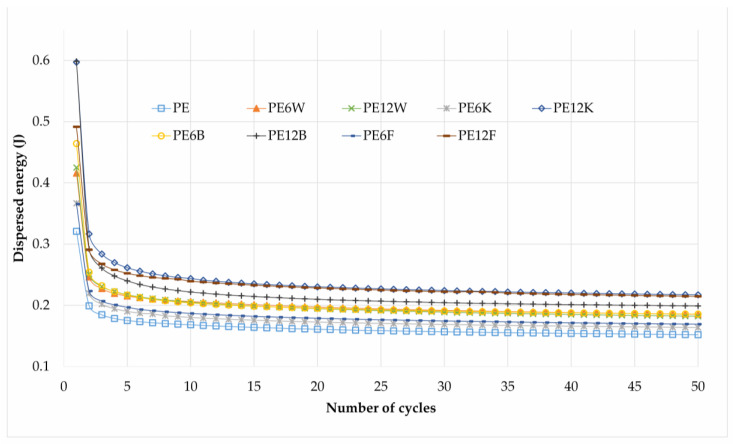
Comparison of dissipated energy as a function of following hysteresis loops.

Composites of bio-polyethylene with natural fibers were tested using load-unload process. As the number of cycles increased, the decrease in dissipation energy could be observed at the same stain level. Its value stabilized after the first few load cycles (Figure 6). The differences between the amounts of dissipated energy can be excellent information to learn about the structure and interrelationships between the matrix and the filler. It can be assumed that in the micro-areas, the adhesive bonds between the fiber and the matrix are in more or less randomly distributed configurations because of their ability to absorb external loads and therefore to approach critical stress states. The first load cycles reveal this phenomenon. They eliminate local, extreme stress areas in the volume of material by successively cracking the critical tension of adhesive joints between components. This interpretation is supported by the fact that the value of dissipated energy gradually decreases in subsequent load cycles. The size and nature of this phenomenon are closely related to the type of adhesion between the matrix and reinforcement as well as to the degree of non-homogeneity of the state of the stress in the composite.

**Figure 7:**
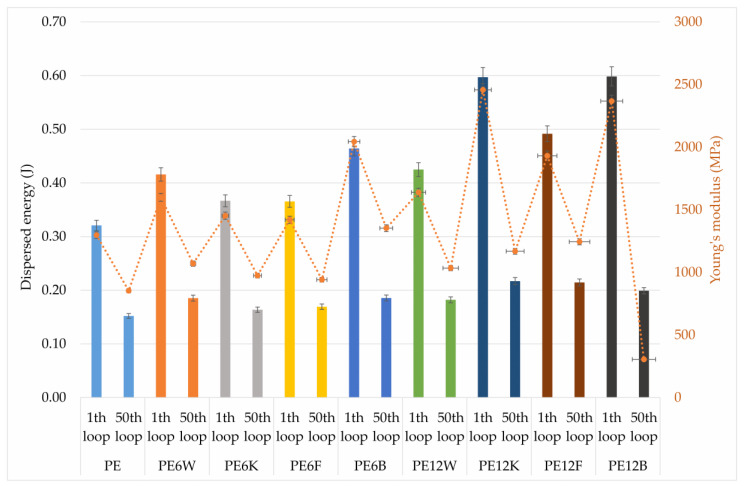
Relation of dispersed energy and Young modulus obtained in first and fiftieth hysteresis loop.

Figure 7 shows the dissipation energy value in the first and fifth cycle of load-unload. Each of the additives raised the dissipation energy relative to the unreinforced material. Doubled content of wood particles in compositions did not change the dissipation energy. Basalt fiber and coconut fiber materials were characterized by the highest dissipation energy, especially in the first cycle, which, according to the theory mentioned above, may suggest a large amount of stress inside the material. Related to natural fibers, it seems to be the most advantageous to use flax fibers, because increase in the fiber content from 6 to 12% by weight did not bring about such large changes in the dispersed energy in relation to the other fibers, and the difference in energy dissipation between the first and the fifth cycle was the smallest, which may suggest a lower degree of internal stress.

**Figure 8:**
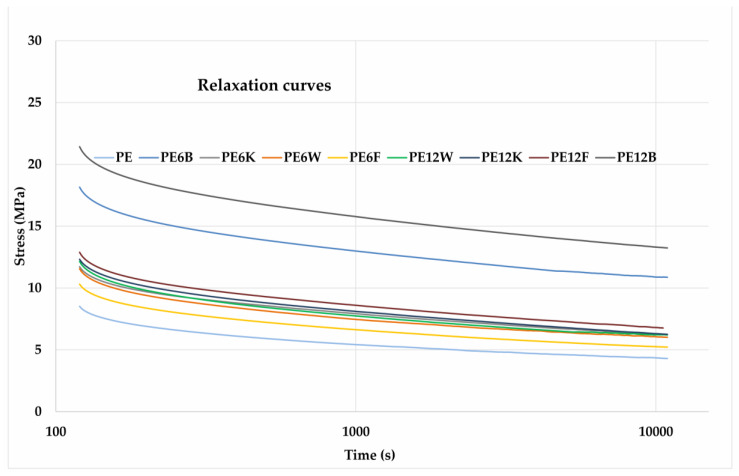
Relaxation short-term relaxation test curves of exanimated composites.

The relaxation process consists of recording changes in force or stress in a specimen during a sustained period of time. When a plastic material is suddenly deformed, high internal stresses are created, which gradually decrease over time, and that reaction is called relaxation. This process occurs both in the glassy and the highly elastic phase. In the glass phase, relaxation occurs by rotating the chain segments (Kuhn segments). In the elastic phase the relaxation is caused by the gradual conversion of the polymer chains into new places. The decay of stress is fast in the beginning and slows down later. This is due to the effect of stress on the segmental rotation time and the time needed to shift to the new position of the macromolecules. time. Relaxation curves are presented in Figure 8.

### 3.2. Thermal Aging and Water Immersion Investigation

The visible effect of chemical changes occurs during heating of the polymers, such as: decrease of molecular weight and emission of low molecular weight gas products. In linear polymeric materials, as a result of degradation, the macromolecular chain is shortened and consequently, the molar mass is reduced. In the polymers with more complicated chain structure, apart from the processes of cracking of the main polymer chain, there are also side group breaks reactions. Thus, the stability, i.e., the durability of the polymers, is strongly dependent on the strength of the bonds. The type and energy of bonds influences both the mechanism of degradation process and its rate.

**Figure 9:**
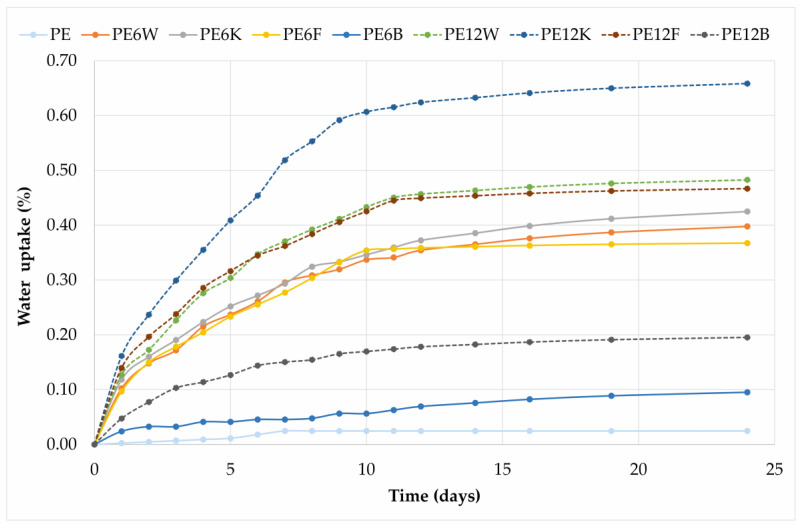
Water absorption of tested composites.

Effect of immersion samples in water is presented in Figure 9. A large water absorption effect of up to about 2 weeks can be seen. This is related to two facts, the first of which is the introduction of fibers that act as capillary and transports water into the material. Second, natural fibers belong to strengthening materials that absorb water, hence the increased absorption effect. After 14 days the water absorption decreased, striving to saturate the composites.

Mechanical properties resulted after water and thermal ageing are compared in Table 5. The interaction of water with polymers is characterized by several mechanisms. Examples include hydration—i.e., the binding of macromolecules to water; formation and stabilization of ordered molecular structures, formation or destruction of hydrogen bonds. The resistance of composites to water depends on the polymer, which is the matrix of the composite, the filler, and the method of connection of the matrix with the filler. In the composite-water system, both the chemical and physical changes mentioned above can occur. Further changes under the influence of water include swelling of the material caused by the migration of water molecules into their interior of the material. In front of the swelling front, tensile stresses arise, which can cause the formation of microcracks [2]. The analysis of mechanical properties carried out for materials after 30 days of immersion in water did not show any significant changes. For materials with a filler content of 6% by weight, a several percent increase in strength was noticed. Strength properties for materials with a higher degree of filling decreased, which suggests that together with the increase of the filling and water content in the material increased, the new resulting stress worsen the connection between the components.

Apart from the chemical or physical action of the same factor, the durability of the material used in the environment is significantly influenced by additional physical or mechanical factors. Temperature is a factor that not only affects the acceleration of possible chemical reactions between the environment and plastic, but also accelerates the diffusion of liquids or gases. Both of these phenomena reduce the mechanical strength of the material and increase its susceptibility to other physical and chemical factors. In addition, by increasing the temperature, regardless of the presence of aggressive factors, it reduces the immediate strength of thermoplastics. The variability of working conditions is a factor significantly reducing the durability of a material. For example, temperature changes due to the high thermal expansion of plastics cause deformations and lead to fatigue damage. Therefore, cyclical changes in environmental temperature are very harmful than the continuous impact of elevated temperature. Moreover, constant contact with the liquid is less harmful than repeated wetting and drying of the material, which alternately causes swelling and shrinkage, which leads to faster destruction as during changes in temperature [48].

These phenomena were observed during accelerated ageing, where the materials were exposed to elevated temperature and high humidity. First, ageing effect in neat bio-polyethylene caused an increase in strength as well as modulus by about 10% and substantial reduction in strain from over 100 to 4% probably as an effect of developing oxidation process in bio-polyethylene [49]. Bio-polyethylene belongs to semi-crystalline materials which do not absorb water, but higher temperature can lead to build up the degree of crystallinity, affecting the increase in strength properties, which was confirmed in the research. In the case of polyethylene-based composites, accelerated ageing significantly reduced the strength and plastic properties.

Research of water absorption led to making theoretical calculation of diffusion coefficient and its kinetics. Diffusion coefficient, parameters k and n are presented in Table 6. The Fick’s diffusion coefficient (D) was estimated by Equation (2) in the range where the values of percent weight gain were less than 60% of the equilibrium value (*M*m):

(2) $$D=\pi \cdot {\left(\frac{k\cdot h}{4M_{m}}\right)}^{2}$$

where *M*m is the maximum moisture content, *h* is the thickness of the sample, and *k* is the initial slope of a curve of M(t) versus t^1/2^, as it can be seen in Equation (3):

(3) $$k=\frac{M_{2}-M_{1}}{\sqrt{t_{2}}-\sqrt{t_{1}}}$$

**Figure 10:**
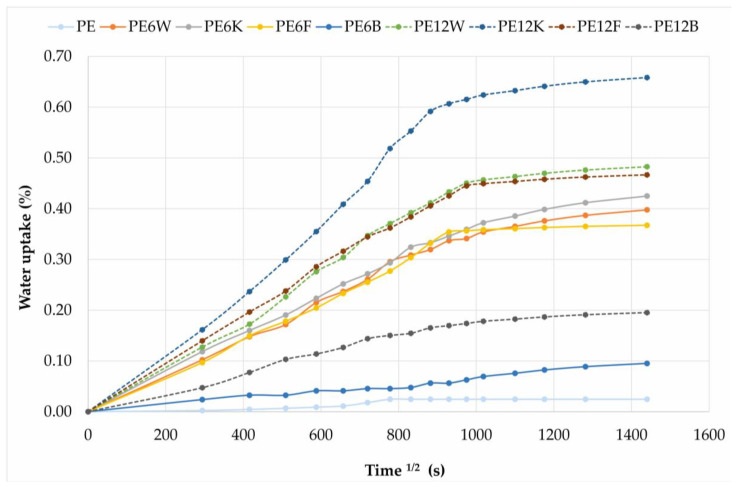
Water absorption curves of different composites as function of square root of immersion time in seconds.

Figure 10 shows water absorption for composites as a function of square root of immersion time. Each point corresponds to the average of five samples. Each of the composites has a similar pattern of water absorption. The initial linear and rapid increase in absorbency is followed by a plateau effect without gain in water absorption representing a Fick’s mode of diffusion.

In general, diffusion behavior can be classified according to the relative mobility of penetrant and polymer segments. There are three types.

Type I or Fick’s diffusion (Equation (2)), in which the diffusion rate is much lower than the mobility of the polymer segment. The balance inside the material is quickly reached and maintained regardless of time.

Type II, in which penetration mobility is much higher than in other relaxation processes. This diffusion is characterized by the development of the border between the swollen outer and inner polymer materials. The boundary moves at a constant speed and the core decreases until the penetration balance of the entire polymer is reached.

Non-Fick diffusion or anomalous type occurs when the penetrator mobility and relaxation of the polymer segment are comparable. It is therefore an intermediate behavior between case I and case II.

**Figure 11:**
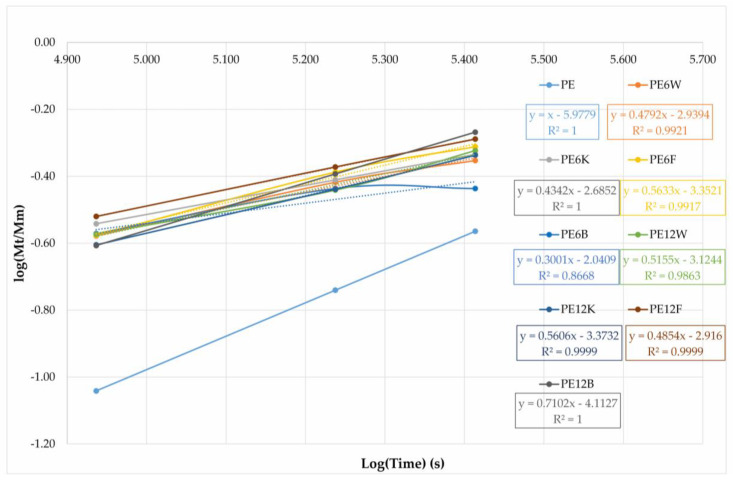
Calculated experimental data to estimate parameter *n* associated with the diffusion mode.

Distinction between these three mechanisms can be done using theoretical considering the shape of the absorption curve, which can be modeled using Equation (4).

(4) $$\frac{M_{t}}{M_{m}}=kt^{n}$$

where Mt is the moisture content, Mm is the moister content at saturation, and *k* an *n* are constant. The parameter *n* is associated with the diffusion mode and takes different values, depending on the specific case: for Fick’s diffusion *n* = 0.5, type II *n* = 1, and for type III (anomalous diffusion) 0.5 < *n* < 1. The values of *n* and *k* can be determined from the slope and intersection of *M*t/*M*m vs. t on a logarithmic graph obtained from experimental data (Figure 11) according to the Equation (5):

(5) $$\log\frac{M_{t}}{M_{m}}=\log\left(k\right)+\mathrm{nlog}\left(t\right)$$

Table 6 summarizes the values of the parameter n that resulted from matching all samples. The *n*-values for composites were close to each other and indicated a value of 0.3–0.7, thus suggested Fick’s diffusion mechanism in composites, but this calculation is devoted to materials that do not absorb water, hence the deviation from the theoretical value can be attributed to the occurrence of other mechanisms in fiber composites such as swelling, weakening of the fiber/matrix interface, micro-cracking of the matrix. Lower values of the parameter *n* were obtained for materials with 6 wt.% of fibers at which the diffusion rate was probably slower than the segment mobility of the polymer.

## 4. Conclusions

The introduction of natural fibers in a small amount of 6% by weight increased the stiffness of the material, without changing its tensile strength. Doubling the fiber content to 12% by weight affected both the strength and stiffness of the material at tensile and bending stresses. From natural fibers, the greatest potential when reinforcing polyethylene was flax fiber, which increased strength properties, ensuring relatively good plastic properties, eliminated the brittle cracking of the composite in comparison with other tested materials. The introduction of fibers into bio-polyethylene caused the water to be absorbed by the composites, and the strength properties after water and thermal aging are reduced as opposed to the unfilled material. The conducted research allowed for an initial assessment of the effectiveness and mechanisms of strengthening individual natural fibers in composites based on bio-polyethylene, and the further direction of research will be the production of hybrid composites using the potential of natural fibers. Such materials could be a good alternative to glass fiber materials used in elements of rehabilitation equipment for humans or animals, as well as in household and automotive articles.

## Figures and Tables

**Table 1:** Description of tested materials.

Index	Description
PE	100 wt.% Green PE SHC7260 Braskem (Bio-PE)
PE6W	92 wt.% of Bio-PE + 6 wt.% wood flour + 2 wt.% Scona TPPP 9112 FA
PE6K	92 wt.% of Bio-PE + 6 wt.% coconut shell fibers + 2 wt.% Scona TPPP 9112 FA
PE6F	92 wt.% of Bio-PE + 6 wt.% flax fibers + 2 wt.% Scona TPPP 9112 FA
PE6B	92 wt.% of Bio-PE + 6 wt.% basalt fibers + 2 wt.% Scona TPPP 9112 FA
PE12W	86 wt.% of Bio-PE + 12 wt.% wood flour + 2 wt.% Scona TPPP 9112 FA
PE12K	86 wt.% of Bio-PE + 12 wt.% coconut shell fibers + 2 wt.% Scona TPPP 9112 FA
PE12F	86 wt.% of Bio-PE + 12 wt.% flax fibers + 2 wt.% Scona TPPP 9112 FA
PE12B	86 wt.% of Bio-PE + 12 wt.% basalt fibers + 2 wt.% Scona TPPP 9112 FA

**Table 2:** Chemical composition of used fibers.

Wood Flour (wt.%)	Coconut Shell (wt.%)	Flax Fibers (wt.%)	Basalt Fibers (%)
Cellulose: 40–50	Cellulose: 26.5	Cellulose: 71	SiO_2_: 51.6–59.3
Hemicellulose: 15–25	Pentosans: 27.7	Hemicellulose: 18–20	AL_2_O_3_: 14.6–18.3
Lignin: 15–30	Lignin: 29.4	Lignin: 2.2	CaO: 5.9–9.4
Moisture: 8–16	Moisture: 8	Moisture: 10	MgO: 3.0–5.3
-	Solvent extractives: 4.2	Pectin: 2.3	FeO + Fe_2_O_3_: 9–14
-	Uronic anhydrides: 3.5	Wax: 1.7	TiO_2_: 0.8–2.25
-	Ash: 0.6	-	Na_2_O + K_2_O: 0.09–0.13

**Table 3:** Physic–mechanical properties of used fibers [40,41,42,43].

Fibers	Density (g/cm^3^)	Diameter (µm)	Length (mm)	Tensile Strength (MPa)	Tensile Modulus (GPa)	Strain at Break (%)
Wood flour	1.3–2.2	300–500	-	44–90	6–13	2.0–3.0
Coconut shell	1.1–1.3	100	2.0	130–250	4–15	2.0–4.0
Basalt	2.7	13–22	3.2	2800–2900	70–110	2.5–3.5
Flax	1.40–1.45	50–70	1–65	400–1200	40–70	1.8–3.2

**Table 4:** Basic mechanical properties of tested material.

Index	Density, g/cm^3^	Tensile Strength, MPa	Tensile Modulus, MPa	Strain at Break, %	Flexural Strength, MPa	Flexural Modulus, MPa	Impact Strength, kJ/m^2^
PE	0.961 ± 0.002	18.1 ± 0.3	1062 ± 26	>200	19.3 ± 0.5	809 ± 21	unbroken
PE6W	0.976 ± 0.001	18.7 ± 0.5	1974 ± 21	5.6 ± 0.6	23.4 ± 0.6	995 ± 25	13.5 ± 0.1
PE6K	0.968 ± 0.004	18.5 ± 0.6	1596 ± 12	5.1 ± 0.4	24.9 ± 1.1	978 ± 54	16.6 ± 1.6
PE6F	0.969 ± 0.001	19.2 ± 0.8	1508 ± 36	7.0 ± 0.5	24.4 ± 1.5	1065 ± 14	31.7 ± 2.4
PE6B	0.988 ± 0.002	24.7 ± 1.2	2280 ± 15	4.1 ± 0.9	30.1 ± 1.2	1316 ± 16	16.6 ± 0.2
PE12W	1.001 ± 0.002	19.3 ± 0.4	1695 ± 25	5.3 ± 0.7	29.9 ± 0.9	1465 ± 26	13.5 ± 1.1
PE12K	0.988 ± 0.003	18.9 ± 1.1	1389 ± 14	5.4 ± 0.5	30.2 ± 0.7	1190 ± 24	13.0 ± 0.1
PE12F	0.991 ± 0.002	21.3 ± 0.7	2311 ± 34	2.8 ± 0.5	33.6 ± 1.8	1633 ± 15	15.9 ± 0.3
PE12B	1.011 ± 0.001	34.0 ± 1.4	2484 ± 18	4.0 ± 0.7	44.9 ± 2.3	2160 ± 31	17.1 ± 0.1

**Table 5:** Mechanical properties measured after water and thermal ageing.

Index	Properties after Water Incubation			Properties after Thermal Ageing		
	Tensile Strength, MPa	Tensile Modulus, MPa	Strain at Break, %	Tensile Strength, MPa	Tensile Modulus, MPa	Strain at Break, %
PE	21.3 ± 0.1	1210 ± 27	>100	18.7 ± 0.9	1315 ± 26	4.0 ± 0.6
PE6W	21.4 ± 0.1	1818 ± 106	5.3 ± 0.3	15.2 ± 0.1	1644 ± 21	2.3 ± 0.1
PE6K	21.0 ± 0.1	1727 ± 203	5.1 ± 0.7	13.2 ± 0.1	1621 ± 86	1.8 ± 0.1
PE6F	19.3 ± 0.1	1527 ± 19	4.7 ± 0.1	16.6 ± 0.8	1439 ± 23	3.4 ± 0.4
PE6B	27.8 ± 1.2	2134 ± 97	4.0 ± 0.2	20.6 ± 1.2	2745 ± 162	1.8 ± 0.3
PE12W	20.4 ± 0.1	2055 ± 16	3.8 ± 0.5	14.6 ± 1.0	2024 ± 24	2.0 ± 0.3
PE12K	19.9 ± 0.4	1504 ± 36	4.5 ± 0.1	11.8 ± 0.5	1391 ± 25	1.9 ± 0.7
PE12F	21.6 ± 0.3	1935 ± 8	3.7 ± 0.4	16.1 ± 0.6	1813 ± 14	2.6 ± 0.9
PE12B	35.0 ± 1.1	3231 ± 19	2.6 ± 0.1	23.1 ± 0.7	3175 ± 35	3.8 ± 0.4

**Table 6:** Estimated parameters for diffusion coefficient and its kinetics.

Samples ID	D (m^2^/s)	Parameter k	Parameter n
PE	4.0 × 10^−12^	0.00003	1.0000
PE6W	3.2 × 10^−12^	0.0004	0.4792
PE6K	2.5 × 10^−12^	0.0004	0.4342
PE6F	3.2 × 10^−12^	0.0004	0.5633
PE6B	7.8 × 10^−13^	0.00004	0.3001
PE12W	3.5 × 10^−12^	0.0005	0.5155
PE12K	3.2 × 10^−12^	0.0007	0.5606
PE12F	3.1 × 10^−12^	0.0005	0.4854
PE12B	5.8 × 10^−12^	0.0003	0.7102
